# Misophonia symptom severity is linked to impaired flexibility and heightened rumination

**DOI:** 10.1111/bjop.70025

**Published:** 2025-09-13

**Authors:** Vivien K. Black, Kenneth J.D. Allen, Hashir Aazh, Sheri L. Johnson, Mercede Erfanian

**Affiliations:** ^1^ Department of Psychology University of California Berkeley California USA; ^2^ Hashir International Specialist Clinics & Research Institute for Misophonia, Tinnitus and Hyperacusis London UK; ^3^ ESSCA School of Management Lyon France

**Keywords:** affective flexibility, cognitive flexibility, misophonia, rumination

## Abstract

Misophonia is a disorder involving sensitivity to certain sounds and related stimuli. Here, we explore the relationship between misophonia and affective flexibility, which describes cognitive shifting abilities in the face of emotion‐evoking stimuli. The secondary aim of this study is to test the potential association between misophonia and cognitive flexibility, building upon findings from previous research. The third objective is to examine the relationship between misophonia and rumination. One hundred and forty participants completed the Memory and Affective Flexibility Task (MAFT), designed to assess affective flexibility, as well as a battery of self‐report measures to evaluate misophonia severity, cognitive flexibility, and rumination. Results suggested an inverse relationship between affective flexibility as measured by switch accuracy, but not reaction time, and misophonia severity. Cognitive flexibility was also inversely associated with misophonia severity, but was not attributed to task‐based affective flexibility, suggesting two independent constructs both involved in misophonia manifestation. Rumination associated positively with misophonia severity and inversely with cognitive flexibility, but not affective flexibility. Taken together, these findings highlight a unique cognitive profile of misophonia, characterized by rigidity at the psychological level through cognitive inflexibility and rumination, as well as at the executive function level in terms of affective switching difficulties.

## BACKGROUND

Misophonia is a disorder characterized by decreased tolerance to certain sounds, such as orofacial (e.g. chewing or sniffing), non‐orofacial but human‐based (e.g. knuckle cracking), or innocuous environmental sounds (e.g. clock ticking). Among individuals with misophonia, these trigger sounds, and sometimes also visual (misokinesia) or other related sensory stimuli, evoke strong emotional reactions such as anger, irritation, disgust, and (anticipatory) anxiety (Swedo et al., 2022). These emotional reactions are accompanied by autonomic nervous system arousal (de Gee et al., 2024; Edelstein et al., 2013; Kumar et al., 2017; Oszczapinska et al., 2024; Schröder et al., 2019) and altered neural activity in areas including those related to emotion processing, the salience of sensory stimuli and motor control (Eijsker et al., 2021; Kumar et al., 2017, 2021; Schröder et al., 2019). Resulting behavioural responses, such as aggression, anger outbursts, and avoidance, often cause significant impairment in daily life, especially in the case of more severe misophonia (Brout et al., 2018). Despite striking prevalence estimates ranging from 4.6% to 20% (Dixon et al., 2024; Vitoratou et al., 2023; Wu et al., 2014; Zhou et al., 2017), research on misophonia, especially its treatment, is still at its early stages (Rosenthal et al., 2023; Swedo et al., 2022). There is also still debate about the class of disorder into which misophonia should fall, if any, as it is not formally recognized in diagnostic manuals such as the DSM‐5‐TR or ICD‐11. However, it is often grouped under the broader umbrella of decreased sound tolerance (DST) alongside hyperacusis, an intolerance of certain everyday sounds, which are perceived as excessively loud or uncomfortable. Although these conditions can co‐occur and share symptoms, they are fundamentally different and distinguishing between them is critical for accurate diagnosis of misophonia (Aazh, Erfanian, et al., 2022; Danesh & Aazh, 2020; Henry et al., 2022; Jastreboff & Jastreboff, 2001, 2015, 2023; Kula et al., 2022).

Evidence has highlighted the presence of executive functioning deficits in individuals with misophonia in areas such as cognitive control, response bias, emotion regulation, and attentional processing (Daniels et al., 2020; Eijsker et al., 2019; Guetta et al., 2022; Murphy et al., 2024; Simner et al., 2021). Those affected often show a tendency to hyperfocus on certain bothersome sounds (misophonic triggers) and face significant challenges in shifting attention away from these stimuli (Edelstein et al., 2013; Jager, de Koning, et al., 2020). They show selective attention deficits in response to trigger sounds as well as hypervigilant anticipation, including anticipatory anxiety (Murphy et al., 2024; Sanchez & Silva, 2018). This pattern may reflect deficits in attentional set‐shifting, a core feature of cognitive inflexibility, which is defined as the inability to disengage from irrelevant stimuli and redirect attention to information relevant to the task or situation at hand (Horning & Davis, 2012; Uddin, 2021). In support of this idea, there is evidence of higher self‐reported cognitive inflexibility among those with misophonia (Simner et al., 2021).

Cognitive flexibility, which facilitates efficient shifting between mental processes to produce contextually appropriate behavioural responses, follows a protracted developmental trajectory and is frequently impaired in numerous common neurodevelopmental and psychiatric disorders (Dajani & Uddin, 2015; Morris & Mansell, 2018), including obsessive‐compulsive disorder (OCD) (Gruner & Pittenger, 2017), anorexia nervosa (AN) (Miles et al., 2020), autism spectrum disorder (ASD) (Albein‐Urios et al., 2018; Lage et al., 2023), post‐traumatic stress disorder (PTSD) (Popescu et al., 2023), and attention‐deficit/hyperactivity disorder (ADHD) (Zhang et al., 2023). Misophonia shows substantial comorbidity with these conditions, and the presence of such comorbidities relates to the severity of misophonia as well (Andermane, Bauer, Simner, et al., 2023; Erfanian et al., 2019; Kluckow et al., 2014; Rinaldi et al., 2023; Rouw & Erfanian, 2018; Wu et al., 2014). The occurrence of these symptoms suggests potential overlap of neurobiological or psychological mechanisms, and cognitive flexibility is one such candidate mechanism.

It remains, however, unclear whether cognitive inflexibility in misophonia manifests primarily in the face of triggers vs. across a broad range of contexts. There are some indications that the cognitive inflexibility may be broad. For instance, misophonia symptoms are associated with a greater aversion to expectation violation in social contexts, suggesting a broader inflexible application of social standards to others' behaviour (Banker et al., 2022). Furthermore, misophonia has repeatedly been associated with perfectionism (Jager, de Koning, et al., 2020; Jakubovski et al., 2022), which is a process associated with cognitive inflexibility, characterized by a *rigid* pursuit of unrealistic personal goals and standards (Egan et al., 2011). In other words, perfectionism may contribute to misophonia by fostering a tendency to inflexibly identify with negative self‐views when self‐imposed standards are not met (Nguyen & Morris, 2024). Perfectionists show an attentional bias that directs greater focus towards negative information than positive information (Shafran et al., 2002). In the context of misophonia, this may translate to an intensified focus on negatively perceived sounds (Simner et al., 2021). These findings may reflect cognitive inflexibility among individuals with misophonia that extends beyond contexts specifically associated with triggering stimuli.

### Limitations of prior flexibility research and the role of affective contexts

To comprehensively understand cognitive flexibility in misophonia, it is crucial to examine whether deficits shown on self‐report data can also be reflected in a neurocognitive/behavioural task. In this respect, behavioural studies have yet to establish a consistent link between task‐based cognitive flexibility and misophonia. Although self‐report measures provide key observations involved with this disorder, the application of behavioural tasks is important, as this has previously not been successfully established. Such tasks capture unique variance in real‐world behaviour and inform intervention targets. They are also less susceptible to subjective bias and can assess automatic response processes, indicating high reliability at the latent‐variable level (Dang et al., 2020; Dennis & Vander Wal, 2010; Friedman & Banich, 2019; Hohl & Dolcos, 2024; Howlett et al., 2021). We propose that prior inconsistent and/or null results regarding cognitive flexibility in misophonia may reflect several mutually inclusive possibilities.

(A) The first possibility could be inconsistent definitions of cognitive (in)flexibility and (B) methodological and reporting biases. (C) The previous null findings could also be due to cognitive inflexibility being evoked only in the presence of triggers (e.g. orofacial sounds) or other relevant stimuli. For instance, the association between impaired cognitive control and misophonia severity was specific to performance on the Stroop task when confronted with misophonia trigger sounds, with misophonia severity not relating to the Stroop effect when faced with sounds considered to be generally unpleasant (Daniels et al., 2020). This could indicate that specific executive functioning deficits may only be present in certain contexts among individuals with misophonia. (D) The other possibility is that self‐report measures are not reliable proxies for neuropsychological tasks of cognitive flexibility (Howlett et al., 2021). As such, the self‐reported cognitive flexibility among those with misophonia (Simner et al., 2021) may not inform the extent to which those individuals show impairments in set‐shifting or task switching, core components intricately linked to cognitive flexibility (Dajani & Uddin, 2015; Stemme et al., 2007). In other words, self‐report measures tend to emphasize personality‐level traits of flexibility rather than task‐specific performance (Buchanan, 2016). For instance, despite reduced cognitive flexibility reported in questionnaires (Simner et al., 2021), individuals with misophonia do not appear to perform differently on the Wisconsin Card Sorting Task (WCST) (Abramovitch et al., 2023), a task in which perseverative responses/perseverative errors are often considered to reflect cognitive inflexibility (Miles et al., 2021). (E) Finally, the null results may be due to the use of only emotionally neutral stimuli in tasks such as the WCST, which may be insufficient to evoke impairments in shifting or switching abilities among these individuals. Considering the role of emotion regulation deficits in misophonia (Dixon et al., 2024; Guetta et al., 2022), emotionally evocative stimuli could be necessary to elicit significant inflexibility in behavioural responding during a task. The potential importance of affective stimuli to evoking flexibility deficits can be shown by drawing parallels with previous research in ASD, which has shown a relationship with misophonia in the form of heightened presence of autistic traits (Rinaldi et al., 2023). Task‐switching paradigms revealed that switch costs associated with autism were specific to trials involving switching attention either to or away from emotional stimuli, with directional findings referring to variations in these costs based on the direction of the shift. In other words, these switch costs differ by direction, reflecting distinct cognitive processes engaged by emotional salience (De Vries & Geurts, 2012; Latinus et al., 2019). Cognitive flexibility in response to emotion‐evoking stimuli is referred to as *affective flexibility* (Eckart et al., 2021).

Thus, this theoretical mismatch constitutes the primary rationale for the present study; prior studies focused on general cognitive tasks lacking emotional relevance, while misophonia is evidently affective (Brout et al., 2018; Erfanian et al., 2018; Kumar et al., 2017; Schröder et al., 2019). Hence, in the present study, we use a novel neurocognitive task, the Memory and Affective Flexibility Task (MAFT) (Allen et al., 2025), which incorporates neutral and emotionally arousing stimuli in working memory and affective flexibility trials (Lang & Bradley, 2007).

### Rumination: a key process related to inflexibility, potentially involved in misophonia

Rumination is recognized as a transdiagnostic process associated with cognitive and affective inflexibility (Altamirano et al., 2010; Davis & Nolen‐Hoeksema, 2000; Genet et al., 2013; Morris & Mansell, 2018). Defined as repetitive, self‐focused thought related to negative emotions and their origins, rumination is pervasive across a range of psychiatric disorders (Treynor et al., 2003). Inherently rigid and maladaptive, rumination involves cycling through the same negative thoughts without transitioning to more adaptive cognitive approaches. Inflexibility may manifest in rumination through deficits in the ability to disengage from negative emotional elements of stimuli, resulting in difficulty shifting focus from negative to neutral or positive cognitions (Genet et al., 2013; Koster et al., 2011). In misophonia, rumination may manifest as inflexible, repetitive negative thinking, not only about trigger stimuli and their sources but also involving internally focused thoughts, such as judgements about the misophonic reaction itself. Misophonic individuals tend to ruminate even when no triggers are present, such as by anticipating what trigger sounds may come next or replaying previous triggering situations (Vitoratou et al., 2021). Although people with misophonia do anecdotally report rumination surrounding their misophonic experience (Bernstein et al., 2013; Dozier & Mitchell, 2023; Podoly et al., 2025), it remains unclear to what extent ruminative thinking is pervasive in broader contexts as an overarching cognitive tendency. Thus, in this study, we ask whether this tendency to ruminate extends more generally to daily life, both about misophonia and other unrelated topics. We aim to explore rumination as a potential manifestation of cognitive and affective inflexibility in misophonia, focusing here on three subtypes: perseverative thinking (Ehring et al., 2011), brooding (Treynor et al., 2003), and anger rumination (Sukhodolsky et al., 2001).

### Current study

By employing the MAFT (Allen et al., 2025), the first aim is to evaluate the relationship between affective flexibility performance and the severity of misophonia symptoms. The MAFT, a neurocognitive task, is designed to assess affective flexibility, which may be relevant to individuals with misophonia as well as broader psychopathology (Friedman & Banich, 2019). To quantify the severity of misophonia, we rely on the Selective Sound Sensitivity Syndrome Scale (S‐Five), a validated multidimensional self‐report tool that captures the impact of misophonia across domains such as emotional reactivity, distress, and functional impairment (Vitoratou et al., 2021). We predict an association of self‐reported misophonia severity with affective inflexibility, as indexed by worse performance on the MAFT switch trials in terms of reduced accuracy and slower Reaction Time (RT) (H1).

In addition, we examine the relationship between self‐reported cognitive flexibility and the severity of misophonia, expecting to replicate the findings of Simner et al. (2021) (H2); thus, we predict a positive association between diminished cognitive flexibility and the severity of misophonia symptoms. Furthermore, we evaluate the association between self‐reported cognitive inflexibility and task‐based indices of affective flexibility, specifically switch accuracy and switch RT derived from the MAFT, to examine whether self‐report flexibility aligns with task‐based measures. We hypothesize that self‐reported and task‐based measures of flexibility will be positively correlated, showing a convergence between cognitive and affective dimensions of flexibility (H3).

Additionally, we investigate the association between rumination, misophonia severity, and affective flexibility, given affective inflexibility, specifically the difficulty in disengaging from processing the emotional aspect of negative stimuli, has been linked to increased rumination in daily life (Genet et al., 2013). In particular, we expect to find a positive relationship between increased self‐reported rumination and misophonia severity (H4). Correspondingly, we expect worse switch accuracy and longer switch RT to correspond with greater levels of rumination (H5). As part of our confirmatory analyses, we also examine the relationship between cognitive flexibility and measures of rumination, which has already been established by a large body of research (Morris & Mansell, 2018). Consistent with previous findings, we hypothesize that decreased cognitive flexibility will be positively correlated with increased levels of rumination (H6).

## METHOD

Ethical approval for the study was obtained from the Committee for Protection of Human Subjects at the University of California, Berkeley (CPHS protocol ID 2024‐01‐17038). The study was pre‐registered on OSF (https://doi.org/10.17605/OSF.IO/UCBG4) before the creation of data.

### Participants

The sample comprised 140 participants (mean age = 29.98 ± 6.72 years; 49 females, 91 males) (Table 1). Of those, 128 participants were recruited from Prolific (www.prolific.com), an online study participant pool, and 12 were volunteer participants recruited from misophonia‐related newsletters; specifically, the soQuiet Misophonia Research Pool (www.soquiet.org/pool) and Misophonia International (www.misophoniainternational.com).

**TABLE 1:** Between‐group demographic comparisons of participants with/without ‘significant’ misophonia.

Parameter	Sig‐misophonia	Non‐sig‐misophonia	*t*/*X* ^2^	*p*	Cohen's *d*
	*M* (*SD*)/% (*n*)				
Gender			4.62	.03	
Female	51.43% (18)	29.52% (31)			
Male	48.57% (17)	70.48% (74)			
Age	31.29 (6.91)	29.54 (6.63)	−1.31	.20	−0.26

*Note*: ‘Sig‐misophonia’ refers to individuals with clinically significant misophonia, whereas ‘non‐sig‐misophonia’ denotes those without clinically significant symptoms (*p* ≤ .05). The *t*‐test analysis is based on participants' scores on S‐Five total.

**FIGURE 1:**
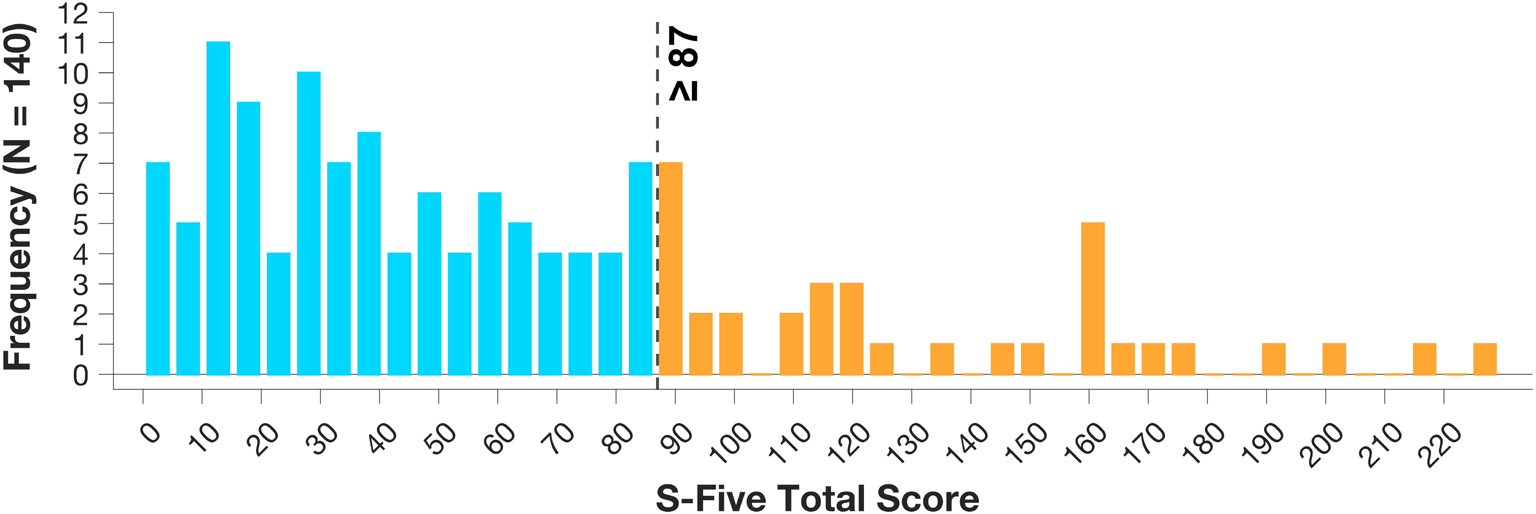
Histogram of S‐Five total score distribution. The dashed vertical line represents the threshold for significant misophonia (87 or above) as indicated by Vitoratou et al. (2023). 25% of the sample (*N* = 35) experienced significant misophonia based on this measure. Scores ranged between 0 and 226 (S‐Five maximum possible score is 250).

Based on the recommended cut‐off score of 87 or above on the S‐Five (Vitoratou et al., 2023), 25% of the total sample (*N* = 35) experienced clinically significant misophonia. Specifically, 18.75% of Prolific participants (*N* = 24) endorsed significant misophonia. This rate is highly similar to the rate of significant misophonia on the S‐Five (18.4%) found by previous research in a general population (Vitoratou et al., 2023). Out of the volunteers identified from misophonia‐related newsletters, 91.67% (*N* = 11) endorsed significant misophonia (Figure 1).

Following current recommendations for transparent sample size reporting (Lakens, 2022), we aimed to recruit the largest practically feasible sample, given the specificity of our target population. We also conducted a separate a‐priori power analysis. Across all analyses, the effect size was chosen to reflect the smallest effect of theoretical relevance in this research context. While Cohen (2013) defined *d* = 0.5 as a medium effect, we align with Correll et al. (2020) in prioritizing theoretical justification and empirical precedent over conventional benchmarks. Although Anderson et al. (2017) warn that published effects may be inflated, our selected effect size represents a conservative but meaningful estimate for outcomes. A‐priori power analysis showed that a minimum sample of 85 participants would provide 80% power to detect a moderate effect size (*d* = 0.15) with an *α* = .05, using MATLAB (2019).

In addition, past studies using behavioural tasks and similar experimental design reported robust effects with a smaller sample size (e.g. Eijsker et al., 2019, *N* = 43, including *N* = 22 with misophonia diagnosis; Daniels et al., 2020, *N* = 79 university students; Simner et al., 2021, *N =* 56 including, *N =* 20 with a misophonia diagnosis). Accordingly, we consider the current sample size sufficient to detect meaningful effects.

### Materials

#### Memory and Affective Flexibility Task (MAFT; Allen et al., 2025)

First, participants completed the Memory and Affective Flexibility Task (MAFT) via Inquisit Web (www.millisecond.com) version 6.1.1.

**FIGURE 2:**
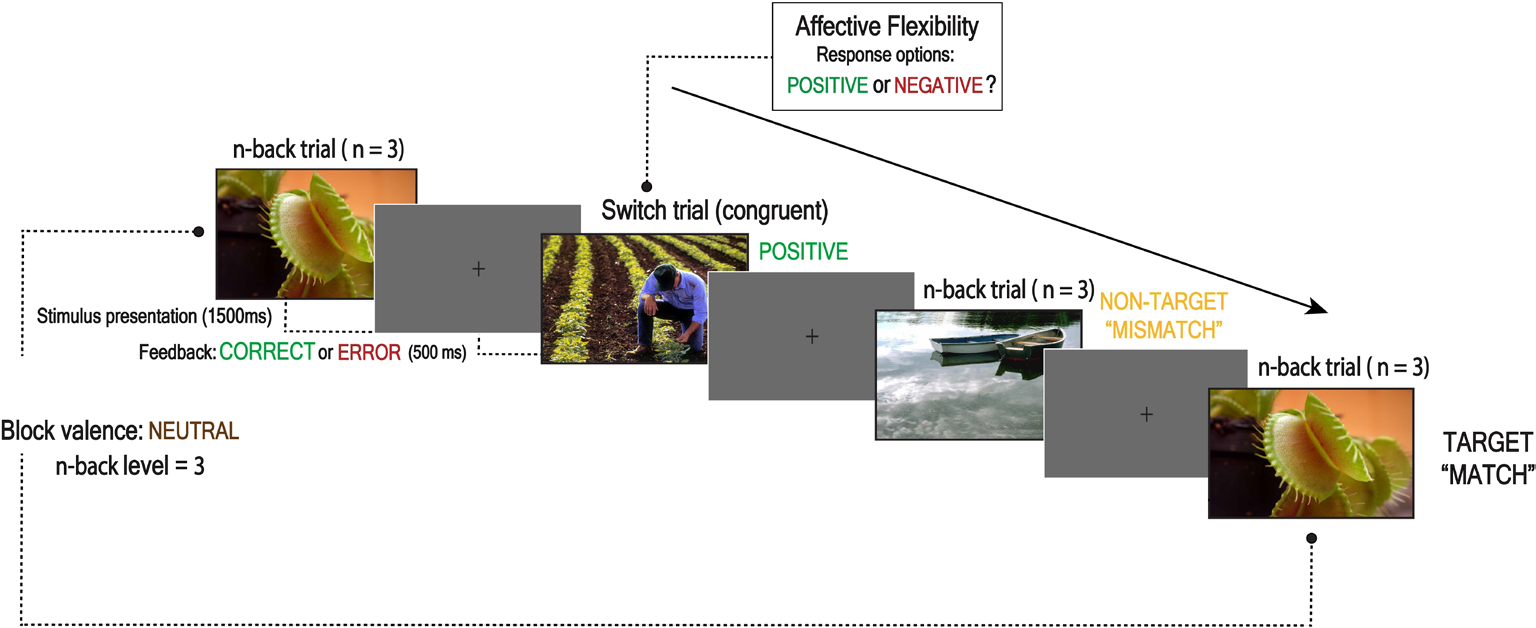
Sequence and timing of stimulus events in the Memory and Affective Flexibility Task (MAFT). A standard block design was used, in which images were presented for 1500 ms with a 500 ms fixation cross as intertrial intervals. Participants judged whether the current image matched or mismatched the image presented 1, 2 or 3 trials prior by pressing ‘A’ for match and ‘L’ for mismatch. After each trial, participants received visual feedback indicating the accuracy of their response, displayed as either ‘CORRECT’ or ‘ERROR’ on the screen. In switch trials, they assessed the valence of emotional images (positive, negative or neutral) from the International Affective Picture System (IAPS) (Lang & Bradley, 2007), by pressing ‘K’ for negative and ‘S’ for positive.

In the MAFT, participants engaged in two types of trials: memory trials and emotional switch trials. Memory trials required participants to determine whether the current image matched one shown *N* trials earlier, measuring working memory. Emotional switch trials, randomly interspersed within the task, required participants to rapidly evaluate whether an image was positive or negative, measuring affective flexibility. The working memory trials were based on the widely used *N*‐back task (Owen et al., 2005), designed to measure behavioural responses to emotional and non‐emotional stimuli. Switch trials assessed affective flexibility, as they required participants to shift from a memory‐based response to evaluating emotional valence of images, reflecting task switching in the context of affective stimuli. For the current study, only switch accuracy and switch RT were of interest (Figure 2).

The MAFT consisted of 198 trials organized into nine experimental blocks, with participants completing three valence‐specific blocks (positive, negative and neutral) for each level of *N*‐back difficulty (*N* = 1, 2 and 3). Each block included 20 + *N* trials to account for initial ‘mismatch’ stimuli before the first potential ‘match’ target. Prior to the experimental blocks, participants completed two practice blocks with 500 ms trial‐level feedback, repeating them until they achieved at least 70% accuracy on *N*‐back trials at *N* = 1 (11 trials) and *N* = 2 (12 trials). Approximately 60% of trials within each block were *N*‐back trials (12 + *N* per block), consisting of four ‘match’ trials and the remainder as ‘mismatch’ trials, with emotional valences congruent with the block type. The remaining 40% (~8 trials per block) were switch trials, which alternated between emotional valences and were presented in a pseudo‐randomized order. Stimuli were drawn without replacement from the International Affective Picture System (IAPS) image pool (Lang & Bradley, 2007), with negative and positive image sets matched on standardized arousal and valence ratings (negative: arousal *M* = 5.94 ± 0.77; valence *M* = 7.22 ± 1.04; positive: arousal *M* = 5.22 ± 1.02; valence *M* = 7.15 ± 0.79), and neutral images selected for low arousal (*M* = 2.88 ± 0.57) and intermediate valence (*M* = 4.98 ± 0.30) (Figure 2).

Performance metrics included accuracy, calculated as the proportion of correct ‘match’ hits and ‘mismatch’ rejections for *N*‐back trials and the proportion of correctly classified positive and negative stimuli for switch trials, as well as RT, calculated as the mean response time for correct trials, consistent with prior research (Eckart et al., 2021; Malooly et al., 2013). Neutral stimuli in switch trials were analysed for interpretive bias, with the proportion categorized as negative serving as an index. Neutral stimuli were predominantly classified as positive (*M* = 0.64 ± 0.11), providing insight into the evaluation of stimuli without salient emotional content.

In memory trials, participants responded by pressing ‘A’ for a match and ‘L’ for a mismatch, while in switch trials, they judged emotional valence, pressing ‘K’ for negative and ‘S’ for positive. Recent findings suggest that RT may not be specific to trial type, as RT indices are highly correlated across tasks, indicating this metric may reflect general motor or cognitive processing speed rather than task‐specific variables (Allen et al., 2025). Although both accuracy and RT were pre‐registered for the switch task, recent evidence highlights accuracy as the more meaningful measure of affective flexibility (Allen et al., 2025); therefore, both metrics are reported here.

#### Selective Sound Sensitivity Syndrome Scale (S‐Five; Vitoratou et al., 2021)

Misophonia symptom severity was assessed using the 25‐item S‐Five (Vitoratou et al., 2021). The S‐Five scale is designed to assess five different dimensions of misophonia severity: externalizing, internalizing, impact, threat, and outburst, as well as overall severity (total score). Participants are asked to respond to questions (e.g. ‘The way I react to certain sounds makes me wonder whether deep inside I am just a bad person’ and ‘The way I feel/react to certain sounds will eventually isolate me and prevent me from doing everyday things’) on a 10‐point scale, ranging from (0) for not at all true to (10) for completely true. The maximum possible score on the S‐Five is 250. A score of 87 or above indicates significant misophonia as suggested by ROC analysis (Vitoratou et al., 2021). The S‐Five has previously exhibited good internal consistency (*α* ≥ .83) and strong test–retest reliability (ICC ≥ .86) for all factors, as well as good convergent and discriminant validity. In the current study, the S‐Five showed excellent internal consistency (*α* = .96).

#### Detail and Flexibility Questionnaire (DFlex; Roberts et al., 2011)

The 12‐item flexibility subscale of the Detail and Flexibility Questionnaire (DFlex) was included as a self‐report measure of cognitive inflexibility (Roberts et al., 2011). In a previous study, participants classified as having misophonia scored significantly higher for inflexibility on this questionnaire compared to those without misophonia (Simner et al., 2021). Participants rated each item (e.g. ‘I can be called stubborn or single minded as it is difficult to shift from one point of view to another’) on a 6‐point Likert scale ranging from strongly disagree (1) to strongly agree (6). Total scores range from 12 to 72, with higher scores indicating greater levels of inflexibility. This scale has previously demonstrated very high internal consistency (*α* = .93) as well as strong construct and discriminant validity. In the current study, the DFlex flexibility subscale showed good internal consistency (*α* = .83).

#### The Perseverative Thinking Questionnaire (PTQ; Ehring et al., 2011)

Perseverative thinking, a measure of Repetitive Negative Thinking (RNT), was assessed using the Perseverative Thinking Questionnaire (PTQ) (Ehring et al., 2011). RNT describes the transdiagnostic process of unproductive, repetitive thinking, and rumination is one form of RNT (Ehring et al., 2011). Participants were asked to rate how each of 15 statements applied to them in terms of how they typically think about their negative experiences or problems. Participants responded using a 5‐point Likert scale, from never (0) to almost always (4). Example statements include ‘The same thoughts keep going through my mind again and again’ and ‘I can't do anything else while thinking about my problems’. The higher‐order PTQ scale has previously exhibited excellent internal consistency (*α* = .94–.95), as well as good internal consistency for subscales (core characteristics of RNT: *α* = .92–.94; unproductiveness of RNT: *α* = .77–.87; RNT capturing mental capacity: *α* = .82–.90); satisfactory test–retest reliability (total score retest correlation = .69), and good convergent validity (Ehring et al., 2011). In the current sample, the higher‐order PTQ scale also showed excellent internal consistency (*α* = .95).

#### Ruminative Responses Scale (RRS; Treynor et al., 2003)

Brooding rumination was assessed using the 5‐item Brooding subscale of the Ruminative Responses Scale (RRS‐B), which is considered the maladaptive form of rumination and has been implicated in the development of psychiatric disorders, such as depressive symptoms (Treynor et al., 2003). Participants responded using a 4‐point Likert scale ranging from Almost Never (1) to Almost Always (4), rating how often they engage with an indicated thought or mental behaviour when down, sad or depressed. Items included, for example, ‘Think “What am I doing to deserve this?”’ or ‘Think about a recent situation, wishing it had gone better’. This scale has demonstrated acceptable reliability (*α* = .77) with a test–retest correlation of .62 in previous research (Treynor et al., 2003). In the current sample, the RRS‐B showed good internal consistency (*α* = .81).

#### Anger Rumination Scale (ARS; Sukhodolsky et al., 2001)

Anger rumination was assessed using the Anger Rumination Scale (ARS) (Sukhodolsky et al., 2001). The ARS comprises 19 items designed to measure the inclination to mentally engage with past episodes or feelings of anger. Participants are asked to rate each of the 19 items on a Likert scale ranging from Almost Never (1) to Almost Always (4), with higher scores indicating heightened anger rumination. Example items include statements like ‘Memories of even minor annoyances bother me for a while’ and ‘I re‐enact the anger episode in my mind after it has happened’. The ARS includes four subscales: angry afterthoughts, thoughts of revenge, angry memories, and understanding of causes. The ARS has previously exhibited adequate internal consistency, test–retest reliability, as well as both convergent and divergent validity (Sukhodolsky et al., 2001). In this study, the internal consistency was excellent (*α* = .93).

#### Screening for Anxiety and Depression in Tinnitus‐Hyperacusis‐Misophonia (SAD‐T; Aazh, Hayes, et al., 2022)

The 4‐item Screening for Anxiety and Depression in Tinnitus‐Hyperacusis‐Misophonia (SAD‐T) is a brief measure of anxiety and depression and has been validated in samples with auditory conditions (Aazh, Hayes, et al., 2022). A score of 4 or higher on this measure is considered the threshold for anxiety or depression symptoms. Participants are asked to rate how often they are bothered by the problems described in each item, with statements such as ‘Feeling nervous, anxious or on edge’ and ‘Feeling down, depressed or hopeless’. In previous research, the SAD‐T has shown good internal consistency (*α* = .91) and satisfactory ITC values between 0.76 and 0.84 (Aazh et al., 2024). In the current sample, the SAD‐T showed excellent internal consistency (*α* = .90).

#### Sound Sensitivity Symptoms Questionnaire (SSSQ; Aazh & Kula, 2024)

The Sound Sensitivity Symptoms Questionnaire (SSSQ) is a 6‐item questionnaire designed to measure symptoms of abnormal sound intolerance, specifically hyperacusis and misophonia. Participants are asked to rate how often each of the statements apply to them, with scores on each item ranging from 0 to 3, corresponding to different frequency intervals (e.g. 0–1, 2–6, 7–10 and 11–14 days). Example statements include ‘Pain in your ears when hearing certain loud sounds? Examples: loud music, sirens, motorcycles, building work, lawn mower, train stations’ and ‘Fear that certain sounds may make your hearing and/or tinnitus worse?’. Total scores range from 0 to 18, with a total score of 4 considered to be the threshold for presence of sound sensitivity (Aazh et al., 2024). We included this measure to control for symptoms of hyperacusis, which has a high rate of co‐occurrence with misophonia (Ahmmed & Vijayakumar, 2024; Andermane, Bauer, Sohoglu, et al., 2023). The previous version of SSSQ, which had 5 items, has shown good internal consistency (*α* = .87) as well as convergent and construct validity (Aazh et al., 2024). Here we utilized a new version with six items, which based on data from the authors showed a good Cronbach alpha estimate (*α* = .80), acceptable McDonald's *ω* with values of .80 and test–retest reliability with an *r*‐value of .81 well above Cohen's suggested threshold for high correlation. In the current sample, the 6‐item version showed acceptable internal consistency (*α* = .77) as well as when removing item 4 (which measures misophonia) from the scale (*α* = .71).

### Procedure

We first recruited participants through Prolific (www.prolific.com), an academic research‐focused crowdsourcing platform. Eligibility criteria as screened using the demographic pre‐screen feature on Prolific included age of 18–45, since individuals within this age range generally have similar audiological profiles, allowing control for hearing impairment/other periphery issues (Lee et al., 2012); no hearing loss or hearing difficulties; absence of cognitive impairment or dementia, normal or corrected‐to‐normal vision; fluency in English; and willingness to be exposed to distressing stimuli, considering the graphic nature of images included in the MAFT task. In addition, although no formal screening was used for comorbid psychiatric diagnosis, this was included as an exclusion criterion in the study advert and consent form (phrased as ‘No current psychiatric diagnosis or cognitive impairment’ in the eligibility section).

Eligible participants based on the Prolific pre‐screen filter were shown a study advert briefly describing the procedures and also stating the compensation amount of $6.73 total USD (~€6.56 or ~£5.51). This compensation amount is equivalent to an estimated rate of $9.58 per hour (~€9.34 or ~£7.85) based on the median completion time of 42.06 min. Two participants, recruited from Prolific during the pilot stage of the study, were compensated in the approximate GBP equivalent to 6.73 USD based on the conversion rate at the time of the study.

After recruitment of participants via Prolific, we initiated an email campaign targeting newsletters (mentioned in Participants section) aimed at individuals interested in volunteering for misophonia research. Those potential participants were sent the same study description and inclusion/exclusion criteria used in the Prolific advert. Those who expressed interest were directed to a Qualtrics pre‐screen survey to assess the same criteria (with identical wording of questions used to filter Prolific participants). Volunteer participants recruited from newsletters were not compensated for their time and were made aware of this before participation. This limitation arose from the requirement to exchange identifiable information, such as PayPal account details, which posed privacy and confidentiality concerns.

All participants were directed via URL to Qualtrics (www.qualtrics.com) to provide informed consent. This involved a description of the nature of images they might encounter during the MAFT task, and participants were required to indicate their willingness to proceed knowing they would be exposed to such images. Upon providing consent, participants were redirected from Qualtrics to complete the Memory and Affective Flexibility Task via Inquisit Web version 6.6.1 (www.millisecond.com).

After completing the MAFT, participants were directed back to Qualtrics, where they re‐entered their subject ID (Prolific‐generated for Prolific participants, and Qualtrics‐generated for volunteers), reported their age and gender, and then completed a battery of questionnaires (e.g. S‐Five, rumination questionnaires, including the ARS, PTQ, RRS‐B, SSSQ, SAD‐T and DFlex). Seven attention checks were included in the questionnaire battery, with one per questionnaire, in an Instructional Manipulation Checks format (e.g. ‘Please select “Often” for this question’).

**FIGURE 3:**
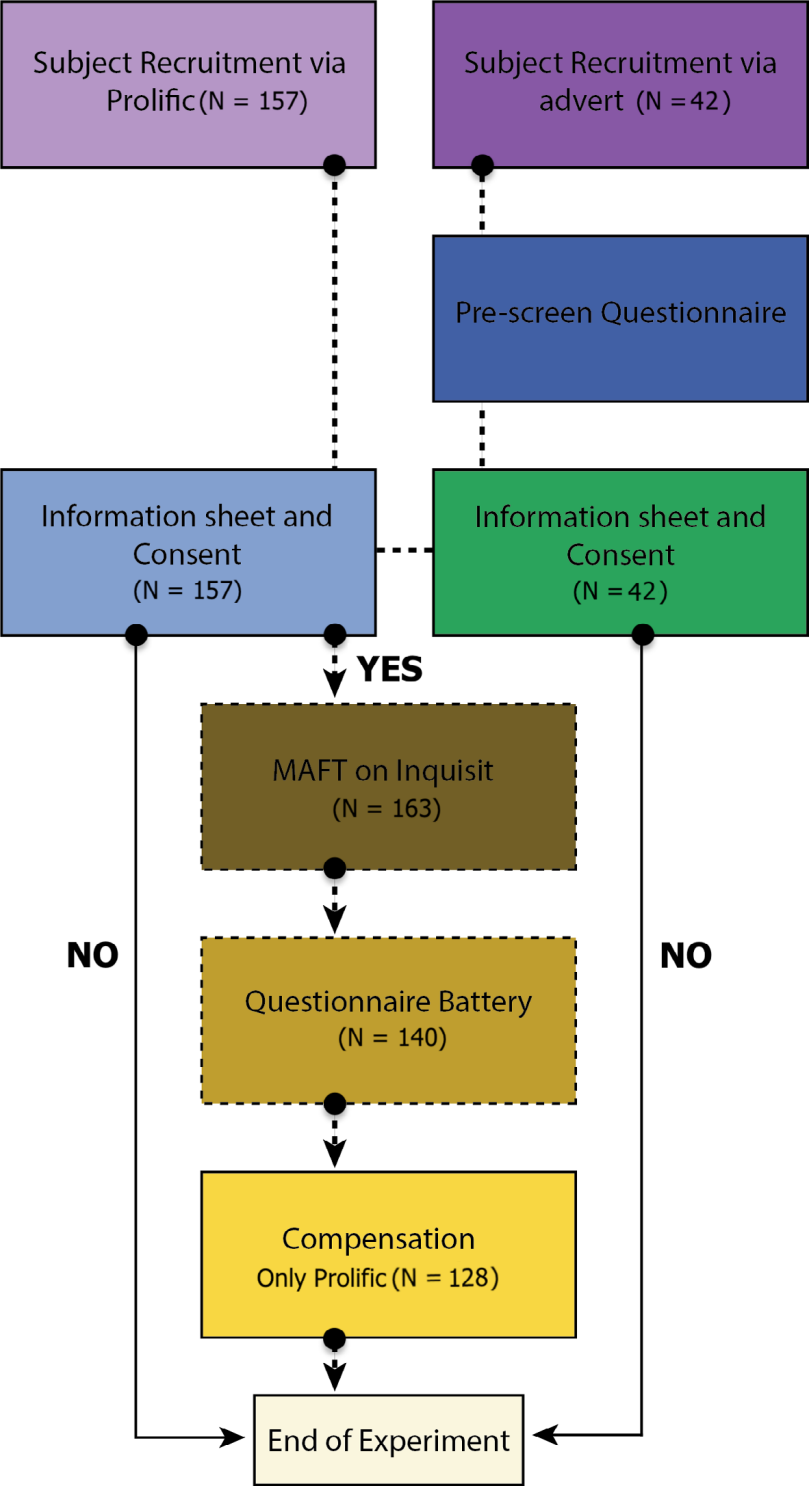
Diagram of online experiment progression. The diagram illustrates the study procedure, detailing each step from initiation to completion, along with the number of participants remaining at each stage. One hundred and ninety‐nine subjects were initially recruited for the study. Of 157 Prolific participants, 142 attempted the MAFT, while 21 of 42 volunteers did so. From 163 total participants, exclusions included incomplete MAFT (*N* = 20), multiple attempts (*N* = 1) and incomplete questionnaires despite completing MAFT (*N* = 2), leaving *N* = 140. The final analysis included 140 participants who successfully completed the study. Only participants providing consent began the experiment. If the participants did not read the information sheet or did not consent, they were debriefed and directed to the end of the experiment.

The entire study, including both MAFT and all questionnaires, took an estimated 40–45 min, with a median study completion time of ~42 min. Upon adequate completion of the battery, Prolific‐recruited participants were provided with a completion code and instructed to return to Prolific to receive compensation through the platform. Participants failing two or more attention checks received a custom code indicating failure and would have been requested to return their submission without compensation; however, no participants failed based on this criterion (Figure 3). Volunteer participants were not redirected upon completion, and the study concluded once they completed all components.

Upon completion, Prolific‐recruited participants' self‐reported demographic data became available for download via Prolific, with optional data including participants' first language, nationality, country of birth, student status and employment status. These data were matched to participants' Inquisit and Qualtrics data using Prolific ID; however, demographic data were not used for analysis purposes in the current study.

A total of 199 participants were recruited for the study, of whom 163 completed the MAFT: 142 from Prolific and 21 from outreach to newsletters. We excluded participants who did not complete the MAFT in full (*N* = 20) or attempted the MAFT task multiple times before completion (*N* = 1) or did not complete the Qualtrics questionnaires in full despite completing the MAFT (*N* = 2), leaving a final sample size of 140 (Figure 3). None of those participants failed two or more of the seven attention checks.

### Independent and dependent variables and covariates

Our key independent variables included affective flexibility (MAFT switch accuracy and switch RT indices), alongside measures of cognitive flexibility (DFlex), perseverative thinking (PTQ), brooding (RRS‐B) and anger rumination (ARS). The dependent variables consisted of the misophonia (S‐Five total score and its five subscales including externalizing, internalizing, impact, threat, and outburst). Possible confounding variables examined in the study were gender, hyperacusis (the scores of the SSSQ), and depression and anxiety (SAD‐T). Gender was included as a covariate due to observed group differences in misophonia severity between male and female participants (see Table 1).

The rationale behind controlling for hyperacusis as a confounding variable was driven by the heightened SSSQ scores, suggestive of hyperacusis, in participants with significant misophonia. This pattern persisted even after excluding the misophonia‐specific item (item 4). This is consistent with previous findings of heightened comorbidity with audiological disorders like tinnitus and hyperacusis (Aazh, Erfanian, et al., 2022). However, misophonia can also occur without audiological abnormalities (Swedo et al., 2022).

Evidence suggests that there is a high prevalence of depression and anxiety in individuals with misophonia (Aazh, Erfanian, et al., 2022; Danesh & Aazh, 2020; Erfanian et al., 2018, 2019; Erfanian & Rouw, 2018; Jager, de Koning, et al., 2020; McKay et al., 2018; Rouw & Erfanian, 2018; Siepsiak et al., 2020; Wu et al., 2014). Hence, we also control for depression and anxiety by incorporating the SAD‐T score as a covariate.

Models controlled for gender, as well as SSSQ scores and SAD‐T scores where appropriate. The inclusion of covariates in each model is explicitly stated in the corresponding results section.

### Data analytic plan

#### Pre‐processing

We pre‐processed MAFT data using Python (Python Software Foundation, 2022), consistent with the methods of Allen et al. (2025). All further statistical analyses were conducted using R version 4.3.1 (Team, 2023) and MATLAB (2019) with an alpha set at .05. Bivariate Pearson correlations were calculated for all measures to assess overlap and correlations between variables.

Implausibly fast responses (<200 ms) and missed responses (>4500 ms), where participants were only allowed to register a response within the 4500 ms trial period, were excluded. Following the approach outlined in Allen et al. (2025), trial‐level outlier RTs with *z*‐scores greater than ±3 (e.g. >3 *SD* from the mean) were trimmed. Participants with a switch omission rate exceeding 0.5 were also excluded from analysis.

Across other measures, we reviewed outliers >3 *SD*. These outliers (S‐Five: two participants >3 *SD* above the mean total score; ARS: one participant >3 *SD* above the mean, and one <3 *SD* below the mean) were retained in the data as they likely reflected natural variation, not measurement error.

Participants were categorized as experiencing clinical misophonia using a threshold score of 87 indicating *significant* misophonia (Vitoratou et al., 2023). Group differences were analysed using *Χ* ^2^ tests for nominal variables and Welch *t*‐tests for continuous variables. Due to a significant difference in gender ratio between misophonia groups, gender was included as a covariate in primary analyses.

#### Model specification (multiple linear modelling)

To address the primary research questions, a series of multiple linear models (MLMs) were conducted to examine (a) the associations between MAFT performance (independent factor) and S‐Five total score and its five subscales (dependent factor) and (b) explore whether these associations remained significant after statistically controlling for DFlex (exploratory analysis). Separate parallel regression models were performed to investigate associations between DFlex, and (c) rumination, including PTQ, RRS‐B, and ARS (both as independent factor), and (d) S‐Five total and its five subscales (dependent factor). This resulted in 41 regression models. Subsequently, we conducted independent regression models to examine (e) the relationships between DFlex (independent factor) and the PTQ, RRS‐B and ARS (dependent factor), resulting in a total of three models. Some of these models were additionally re‐run (only for S‐Five total) after excluding outliers to evaluate the robustness of the results (not driven by outliers), with these instances explicitly noted where applicable.

For the mediation analyses exploring (f) the interaction between rumination and DFlex in predicting S‐Five total, DFlex, ARS, RRS‐B, and PTQ were included as independent variables, with the S‐Five total score as the dependent variable (3 models) (exploratory analysis).

For all models, standardized beta coefficients (*β*) are reported alongside corresponding *t*‐values, and *p*‐values. To mitigate the risk of Type I error (false positives), we also report false discovery rate (FDR)‐corrected *p*‐values (Benjamini & Hochberg, 1995), applying the correction across all the models and correlation analyses.

## RESULTS

### Correlation between study variables

**FIGURE 4:**
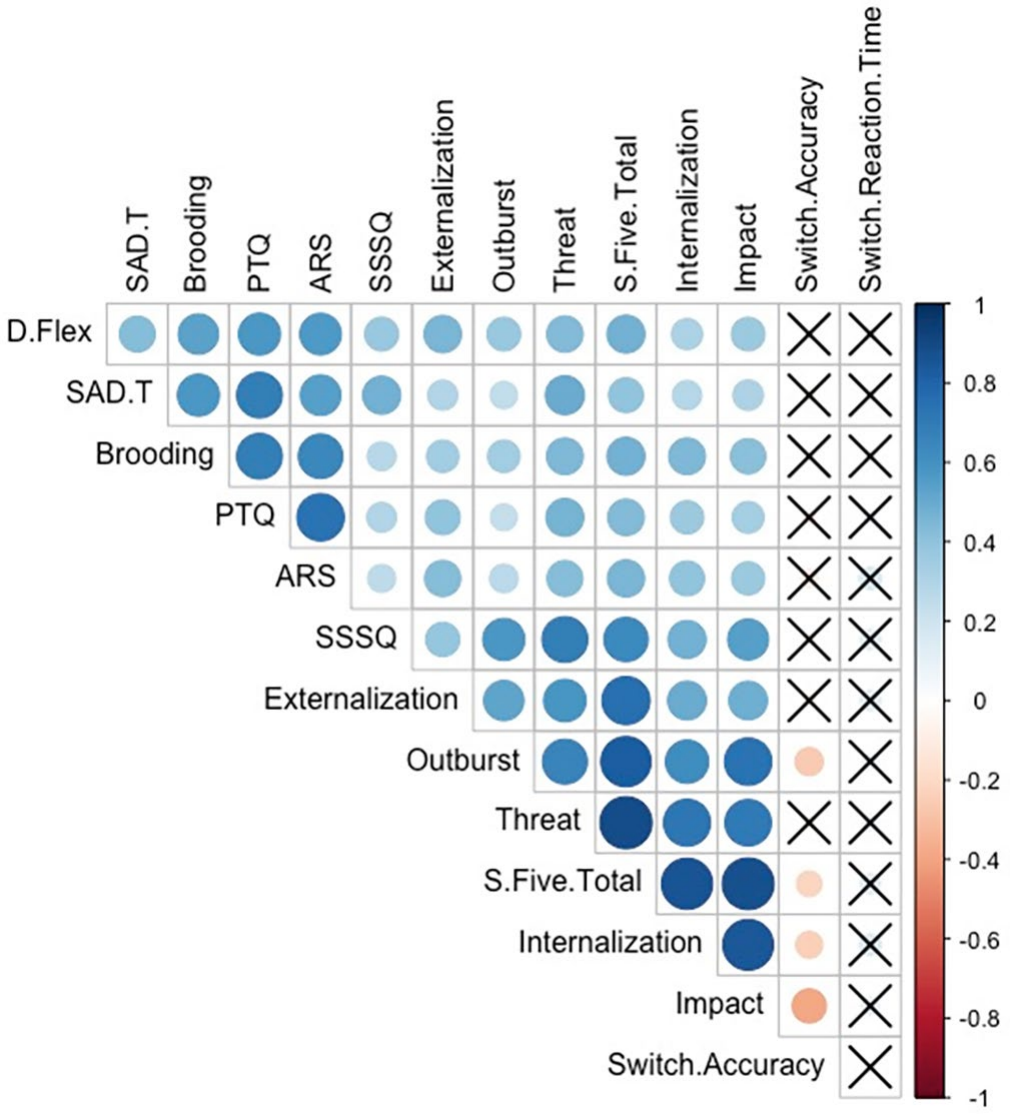
Correlation matrix of variables. The correlation matrix displays Pearson correlation coefficients among all study variables, with relationships represented by colour coding and size scaling. The size and colour gradient of the circles indicate the strength of the correlations, with warm colours (red) denoting negative correlations and cool colours (blue) denoting positive correlations. Circles marked with crosses represent non‐significant correlations (*p* ≥ .05).

To evaluate the degree of overlap among all variables considered for inclusion in the regression models, including independent variables, covariates, and outcome measures, Pearson correlation coefficients were computed. Particular attention was given to identifying highly correlated variables pairs (e.g. *r* > .90) to mitigate potential multicollinearity in the final models, consistent with works by Mitchell et al. (2021) and Erfanian et al. (2021). The variables analysed included the MAFT affective inflexibility indices switch accuracy and switch RT; self‐reported cognitive inflexibility measured by the DFlex; three rumination measures (PTQ, ARS, and RRS‐B); misophonia severity as assessed by the S‐Five total score and its subscales (externalizing, internalizing, impact, threat, and outburst); anxiety and depression symptoms assessed using the SAD‐T; and hyperacusis measured by the SSSQ (Figure 4). Although our findings are consistent with Allen et al. (2025), which suggest that RT scores may primarily reflect individual differences in processing or motor response speed rather than specific effects of task conditions, we nonetheless report RTs alongside accuracy for interested readers.

### Switch accuracy but not switch RT was associated with S‐Five total, internalization, impact and outburst factors

**FIGURE 5:**
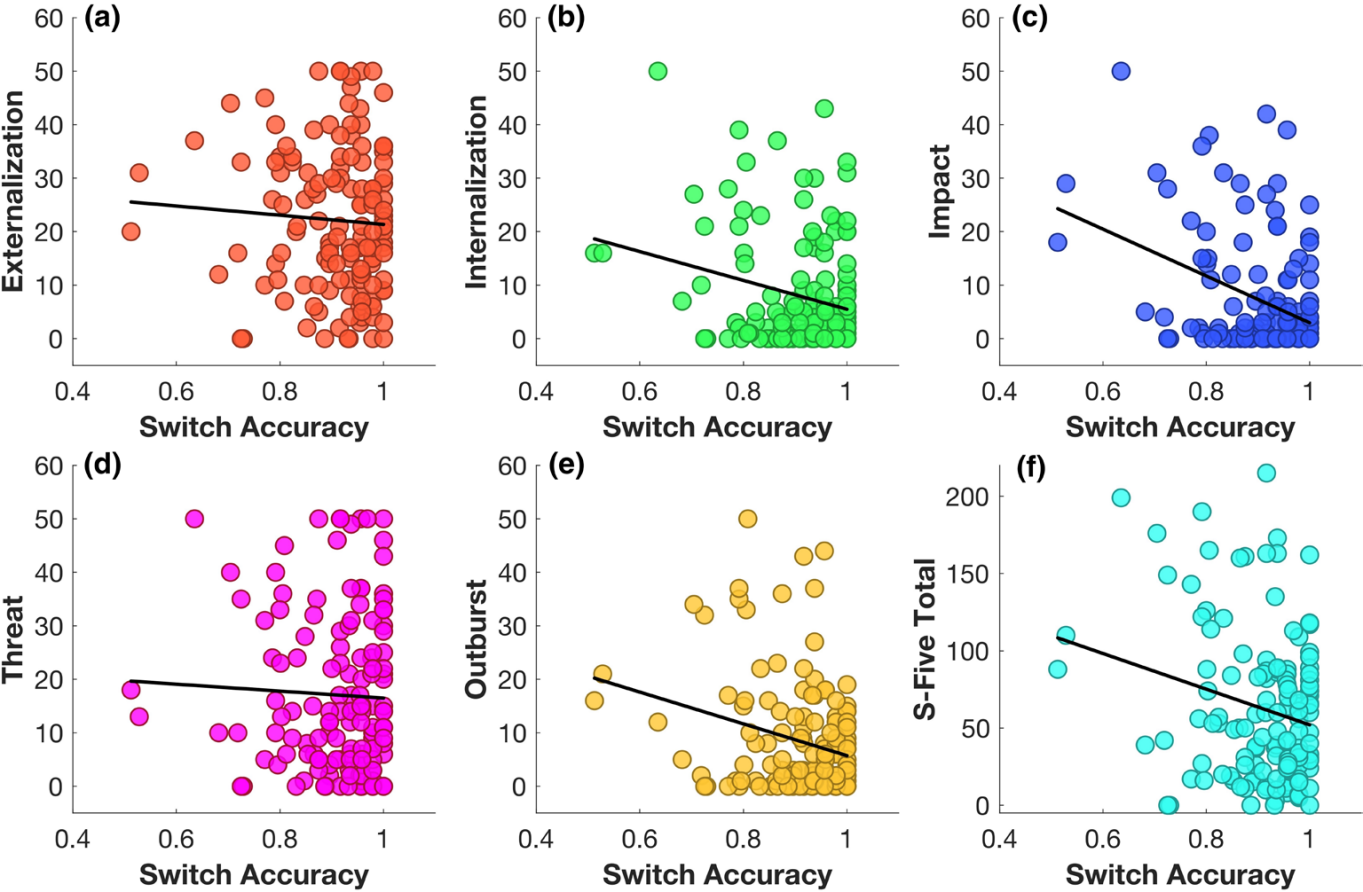
Scatterplot of bivariate analyses. The scatterplots demonstrate the bivariate relationships between MAFT switch accuracy as a proxy of affective flexibility and (a) externalization, (b) internalization, (c) impact, (d) threat, (e) outburst as S‐Five subscales and (f) S‐Five total, reflecting misophonia total severity. The circles represent individual data points, while the diagonal black lines indicate the line of best fit.

Initially, we investigated the relationship of affective flexibility, as measured using switch accuracy and switch RT of the MAFT, with the severity of misophonia (H1). As illustrated in Figure 5, switch accuracy on the MAFT demonstrated a significant inverse relationship with misophonia severity (total score: *β* = −.18, *t* = −2.63, *p* = .01, *p*‐adj = .02), as well as the internalizing (*β* = −.22, *t* = −2.89, *p* = .004, *p*‐adj = .01), impact (*β* = −.36, *t* = −5.31, *p* < .001, *p*‐adj < .001) and outburst domains (*β* = −.23, *t* = −3.26, *p* < .001, *p*‐adj = .004), but not the externalizing (*β* = −.03, *t* = −0.34, *p* = .73, *p*‐adj = .79) or threat (*β* = −.002, *t* = −0.02, *p* = .98, *p*‐adj = .98) domains, controlling for gender, hyperacusis, depression and anxiety as covariates. After excluding the two participants who scored >3 *SD* above the mean S‐Five total score, the relationship between switch accuracy and S‐Five total score remained significant (*β* = −.20, *t* = −2.95, *p* = .004, *p‐*adj = .01).

Given the absence of significant correlations between the switch RT index and misophonia severity (S‐Five total: *r* = .13, *t* = 1.58, *p* = .12, *p*‐adj = .16; Internalizing: *r* = .15, *t* = 1.82, *p* = .07, *p‐*adj = .11; Externalizing: *r* = .14, *t* = 1.66, *p* = .10, *p*‐adj = .14; Impact: *r* = .11, *t* = 1.26, *p* = .21, *p*‐adj = .27; Threat: *r* = .08, *t* = 0.95, *p* = .34, *p*‐adj = .42; Outburst: *r* = .09, *t* = 1.01, *p* = .32, *p*‐adj = .40), or the other variables of interest as determined by Pearson correlation analysis, switch RT was excluded from subsequent multivariate analyses. Although not significant, our findings are consistent with those of Allen et al. (2025).

In spite of the observed association between switch accuracy and misophonia, it could be argued that this relationship reflects a general task‐switching or flexibility impairment rather than involvement of affective‐specific processes, given the absence of a non‐affective cognitive control task to isolate affective demands. To partially address this concern, we conducted follow‐up regression models in which MAFT switch accuracy was entered as a predictor of misophonia severity (S‐Five total score and individual subscales), with the inclusion of DFlex score as a covariate (in addition to the original covariates). The results revealed that switch accuracy remained a significant predictor of S‐Five total score (*β* = −.18, *t* = −2.85, *p* = .005, *p*‐adj = .01), as well as the internalizing (*β* = −.22, *t* = −2.97, *p* = .004, *p‐*adj = .01), impact (*β* = −.36, *t* = −5.50, *p* < .001, *p*‐adj < .001) and outburst (*β* = −.23, *t* = −3.42, *p* < .001, *p‐*adj = .002) subscales, indicating a robust and independent contribution of affective flexibility to misophonia symptoms. In contrast, switch accuracy did not significantly predict the externalizing (*β* = −.04, *t* = −0.49, *p* = .63, *p*‐adj = .70) or threat (*β* = −.01, *t* = −0.08, *p* = .94, *p*‐adj = .95) subscales, consistent with results prior to inclusion of DFlex score as a covariate. This pattern supports the notion of domain‐specific contributions of affective, as opposed to general cognitive, flexibility to misophonia (see the Limitations and future directions section for discussion of the limitations of this approach).

### Participants with significant misophonia scored worse for switch accuracy on the MAFT but did not demonstrate a slower switch RT

**FIGURE 6:**
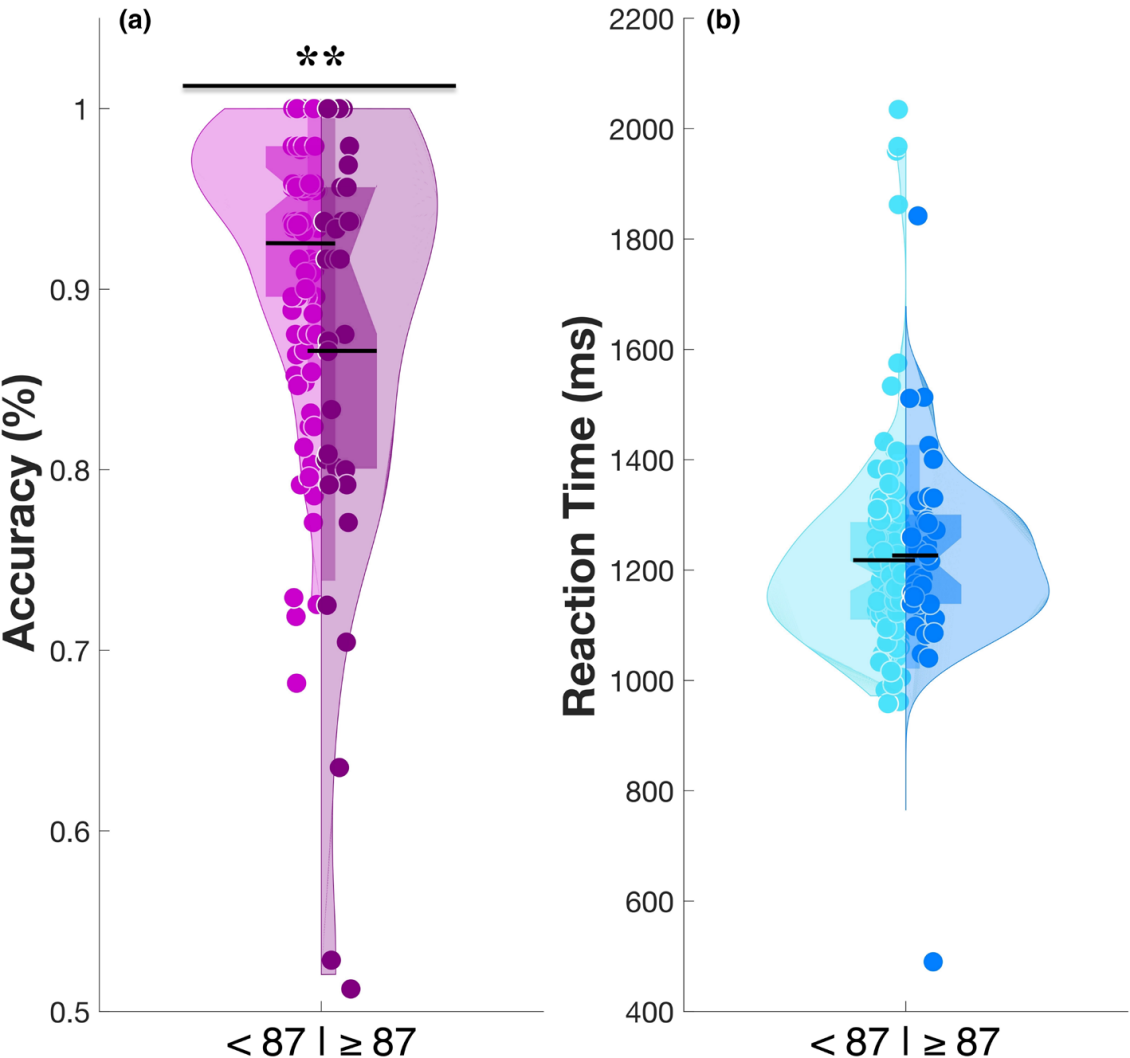
The violin plots of misophonia groups. The figures present the results of Welch *t*‐test comparing between‐group differences in MAFT affective flexibility performance, measured by switch accuracy (a) and switch RT (b), between participants with significant misophonia (scores ≥ 87) and those without. The *X*‐axis represents two groups: Those scoring below and above 87, while the *Y*‐axis displays accuracy as a percentage (%) for switch accuracy and switch RT (ms). Individual data points are represented by single circles. The bottom edge of the thick rectangle represents the 1st quartile (Q1, 0.25), while the top edge represents the 3rd quartile (Q3, 0.75). The lower end of the kernel density curve indicates the 1st percentile (Q 0.01), and the upper end represents the 99th percentile (Q 0.99). The bottom of the long bar illustrates the standard deviation, while the black horizontal line indicates the mean. The top asterisk denotes statistical significance between the groups. *p < .05, **p < .01 and ***p < .001.

Our primary analyses were based on continuous measures of misophonia severity. However, given that a substantial proportion of participants scored below the threshold for clinically significant misophonia (*N* = 105), the sample was also dichotomized using a cut‐off score of 87. This binary classification enabled comparison with prior research, which often relies on threshold‐based criteria to define clinically relevant misophonia (Vitoratou et al., 2023). Additionally, it offers a clinically interpretable benchmark and enables mapping of potential differences between the two groups who fall below and above the threshold, including those who are more symptomatic. Switch accuracy and RT were then compared between those without clinically significant misophonia (<87) and those who met the threshold for significant misophonia (≥87). As shown in Figure 6, we found a significant between‐group difference in mean switch accuracy, with individuals classified as experiencing significant misophonia showing worse accuracy on the MAFT switch trials, with a medium effect size (*t* = 2.61, *p* = .01, *d* = 0.67). No significant difference, however, was observed comparing the two groups' switch RT data (*t* = −0.13, *p* = .90, *d* = −0.03), in line with our continuous‐scale findings. This demonstrates that the relationship between switch accuracy and misophonia is also evident dichotomously when comparing groups based on the S‐Five cut‐off threshold.

The group‐based findings mirrored those observed in continuous models, lending further robustness to the observed associations. Yet, given the unequal group sizes, these findings should be interpreted with caution and viewed as complementary rather than confirmatory.

### S‐Five total and all its subscales were associated with decreased cognitive flexibility score on Dflex when controlling for confounders

**FIGURE 7:**
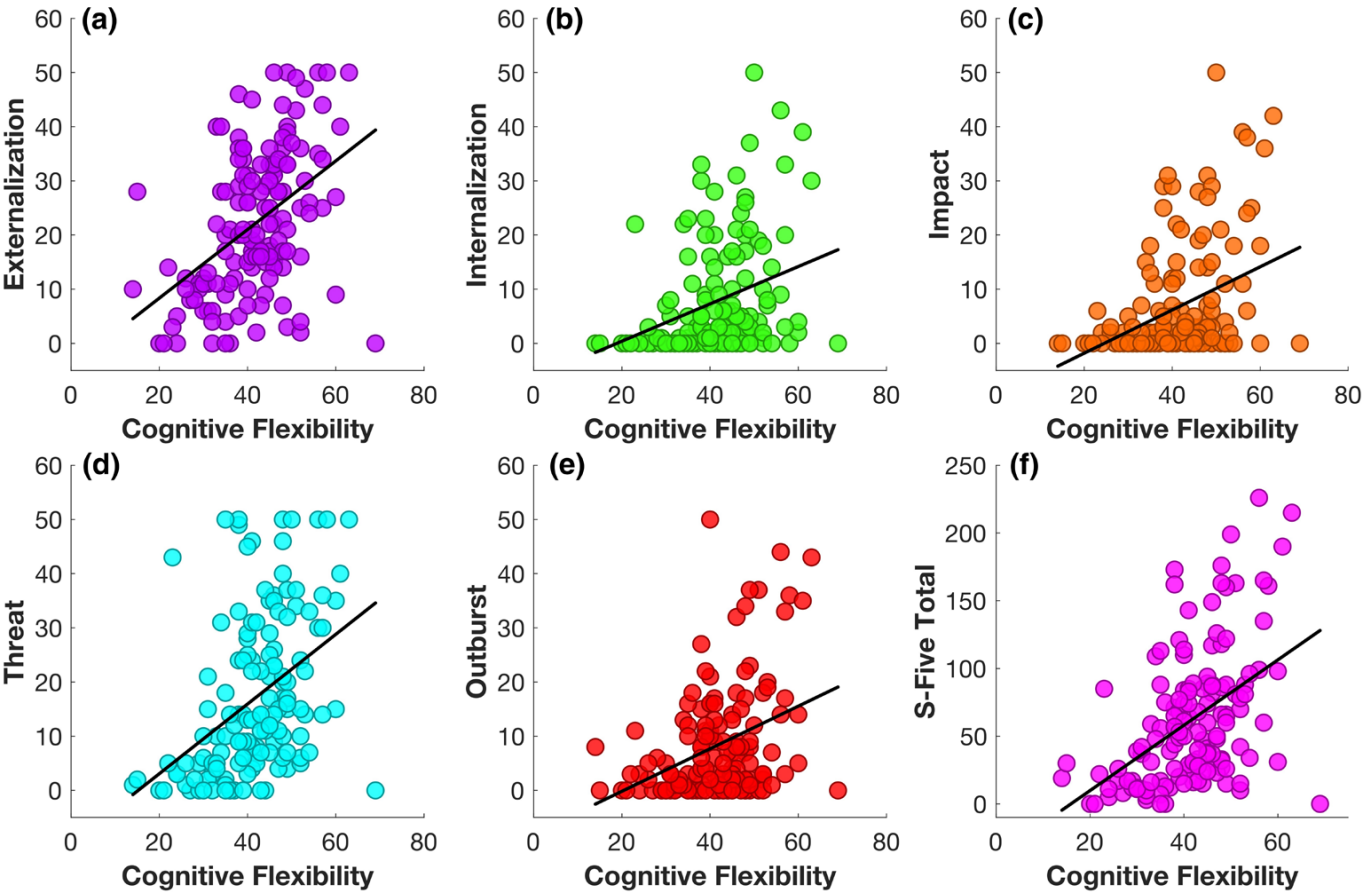
Scatterplot of bivariate analyses. The scatterplots show the bivariate relationships between DFlex representing cognitive flexibility and (a) externalization, (b) internalization, (c) impact, (d) threat, (e) outburst as S‐Five subscales and (f) S‐Five total, reflecting misophonia total severity. The circles represent individual data points, whereas the diagonal black lines show the line of best fit.

To further explore whether impaired mental flexibility potentially extends beyond the behavioural task and can be extrapolated to broader contexts, we tested the replicability of Simner et al. (2021) who demonstrated impaired self‐reported cognitive flexibility in people with misophonia. As shown in Figure 4, Pearson correlation analysis established a significant moderate relationship between S‐Five score (total and all five factors) and DFlex flexibility subscale score, supporting H2. When controlling for gender, hyperacusis, depression and anxiety as covariates, regression models revealed a significant relationship of cognitive inflexibility score with S‐Five total: *β* = .26, *t* = 3.44, *p* < .001, *p*‐adj = .002; externalizing: *β* = .38, *t* = 4.42, *p* < .001, *p*‐adj < .001; impact: *β* = .19, *t* = 2.27, *p* = .02, *p*‐adj = .04; and outburst: *β* = .21, *t* = 2.53, *p* = .01, *p*‐adj = .02. However, DFlex score did not account for significant variance on the internalizing domain of the S‐Five when the aforementioned covariates were included (*β* = .16, *t* = 1.78, *p* = .08, *p*‐adj = .11) and similar results were observed for threat (*β* = .15, *t* = 2.00, *p* = .047, *p*‐adj = .08). Further, the relationship between DFlex and total misophonia severity remained significant (*β* = .23, *t* = 2.98, *p* = .003) when removing the two participants from the analysis who scored beyond the S‐Five total score outlier threshold of 3 *SD* beyond the mean. Integrating these results, our findings indicate that misophonia is significantly associated with self‐reported cognitive inflexibility on a continuous scale. These outcomes not only corroborate but also expand upon the findings of Simner et al. (2021) (Figure 7).

### Neither switch accuracy nor switch RT of MAFT was associated with score on DFlex

Our findings indicated no significant correlation between affective flexibility (MAFT) and self‐reported cognitive flexibility measured by the DFlex (switch accuracy: *r* = .01, *t* = 0.17, *p* = .87, *p*‐adj = .9; switch RT: *r* = .07, *t* = 0.83, *p* = .41, *p*‐adj = .48), in contrast to H3. These results suggest that while misophonia is linked to cognitive inflexibility, the observed relationships are likely independent of the mechanisms captured by switch accuracy‐based measures of affective flexibility in the MAFT paradigm. These findings highlight the notion that task‐based and questionnaire‐based measures may overlap conceptually but likely represent distinct psychological constructs. While DFlex scores tap into participants' perceived cognitive inflexibility, it is important to note that DFlex is a self‐report measure. It gauges general cognitive flexibility as a broad, trait‐like tendency, without directly evaluating task‐switching. As such, it does not function as a cognitive flexibility task which is designed to measure moment‐to‐moment switching performance, limiting our ability to draw conclusions about domain‐general cognitive flexibility. In addition, Dang et al. (2020) suggest that such weak or absent correlations may be related to the poor reliability of some behavioural measures and the involvement of different response processes. However, findings from Abramovitch et al. (2023) support our observation, as they reported no significant differences between individuals with misophonia and controls on the WCST, which is an emotionally neutral (non‐affective) flexibility task.

### Self‐reported rumination was associated with total score of S‐Five, and effects persisted when controlling for gender, SAD‐T and SSSQ

As shown by Figure 4, Pearson correlation analyses revealed moderate positive associations between misophonia severity (including all S‐Five subscales) and all three forms of rumination: perseverative thinking (PTQ), brooding (RRS‐B) and anger rumination (ARS), providing evidence in support of H4. We then used regression models to explore the relationship between these three forms of rumination and misophonia severity while accounting for the influence of potential confounding variables: gender, hyperacusis, depression, and anxiety.

**FIGURE 8:**
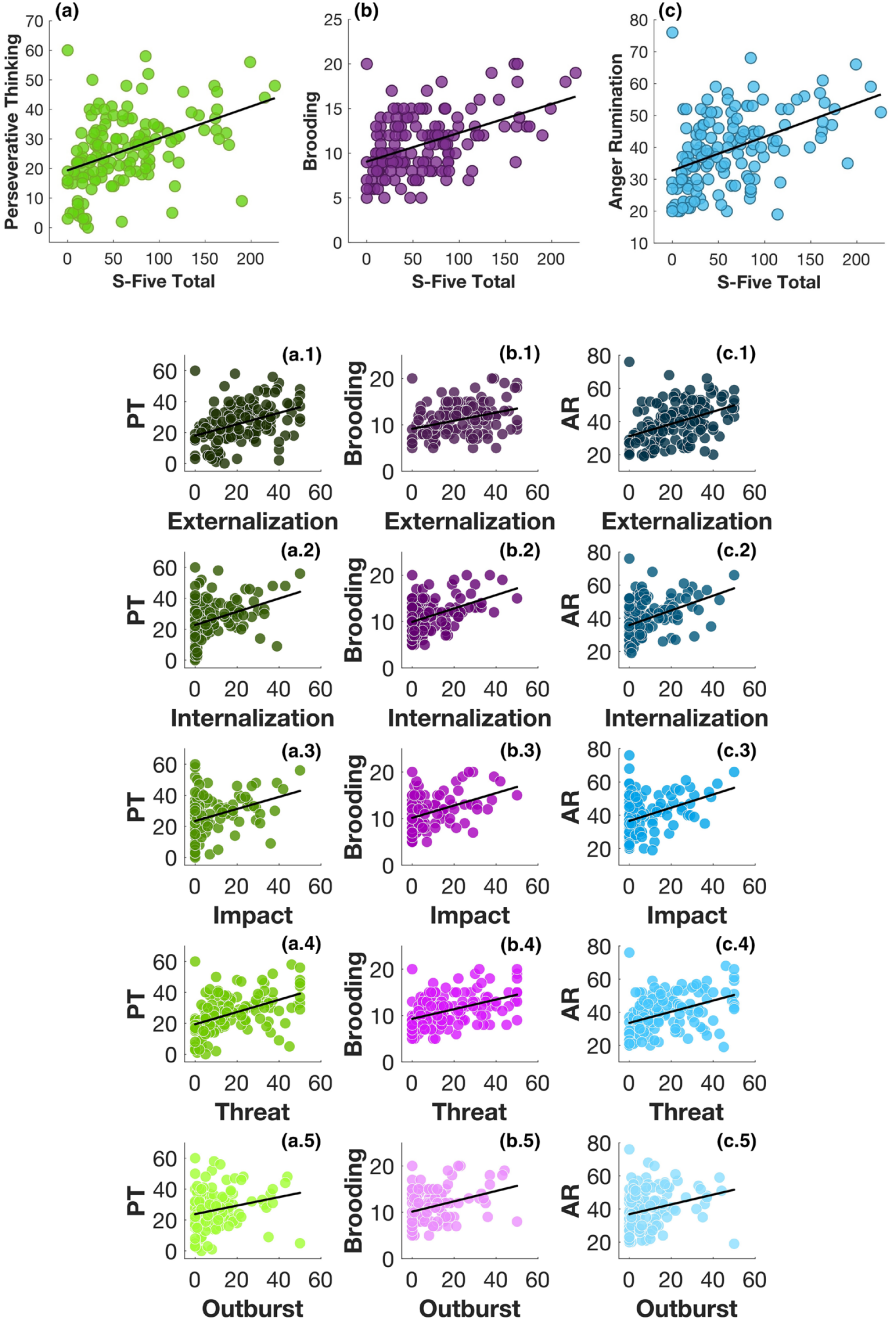
The scatterplots illustrate the bivariate relationships between S‐Five total, reflecting misophonia severity (a–c) and its subscales: (1) externalization, (2) internalization, (3) impact, (4) threat and (5) outburst with (a.1–a.5) perseverative thinking (PT), (b.1–b.5) rumination and (c.1–c.5) anger rumination (AR). The circles represent individual data points, while the diagonal black lines indicate the line of best fit.

Using a separate regression equation for each rumination questionnaire (three total, shown in Figure 8), each subtype of rumination remained a significant predictor of misophonia severity (measured by S‐Five total) when controlling for gender, hyperacusis, and depression/anxiety (PTQ: *β* = .36, *t* = 4.05, *p* < .001, *p*‐adj < .001; RRS‐B: *β* = .36, *t* = 4.65, *p* < .001, *p*‐adj < .001; and ARS: *β* = .33, *t* = 4.37, *p* < .001, *p*‐adj < .001), and also when excluding participants who reached outlier scores on the S‐Five (PTQ: *β* = .35, *t* = 3.92, *p* < .001, *p*‐adj < .001; RRS‐B: *β* = .32, *t* = 4.04, *p* < .001, *p*‐adj < .001) and the one ARS outlier (*β* = .36, *t* = 4.81, *p* < .001, *p*‐adj < .001).

In more details, perseverative thinking was associated with internalization (*β* = .35, *t* = 3.46, *p* < .001, *p*‐adj = .002), externalization (*β* = .41, *t* = 3.98, *p* < .001, *p*‐adj < .001), impact (*β* = .27, *t* = 2.77, *p* = .006, *p*‐adj = .01) and threat (*β* = .28, *t* = 3.29, *p* = .001, *p*‐adj = .004), but not outburst (*β* = .18, *t* = 1.81, *p* = .07, *p*‐adj = .11). We observed similar pattern for brooding and internalization (*β* = .42, *t* = 4.76, *p* < .001, *p*‐adj < .001), externalization (*β* = .27, *t* = 2.80, *p* = .006, *p*‐adj = .01), impact (*β* = .35, *t* = 4.14, *p* < .001, *p*‐adj < .001); threat (*β* = .23, *t* = 2.97, *p* = .004, *p*‐adj = .008) and outburst (*β* = .30, *t* = 3.59, *p* < .001, *p*‐adj = .002). The same pattern continued in anger rumination with internalization (*β* = .34, *t* = 3.98, *p* < .001, *p*‐adj < .001); externalization (*β* = .38, *t* = 4.30, *p* < .001, *p*‐adj < .001); impact (*β* = .27, *t* = 3.25, *p* = .001, *p*‐adj = .004); threat (*β* = .21, *t* = 2.83, *p* = .005, *p*‐adj = .01); and outburst (*β* = .19, *t* = 2.23, *p* = .03, *p*‐adj = .05) (Figure 8).

These results suggest that rumination, not limited to one subtype, is positively associated with misophonia severity and all five dimensions of misophonia. Although both rumination and misophonia are associated with anxiety, depression and hyperacusis (Figure 4), these findings imply that rumination explains variance in misophonia over and above the variance explained by anxiety/depression, hyperacusis and gender.

### Self‐reported rumination was unrelated to MAFT switch accuracy or switch RT, but linked to DFlex

As demonstrated in Figure 4, Pearson correlation analysis established no significant relationship between switch accuracy any of the three rumination subscales: PTQ: *r* = −.07, *t* = −0.85, *p* = .40, *p*‐adj = .48; RRS‐B: *r* = −.02, *t* = −0.22, *p* = .83, *p*‐adj = .88; ARS: *r* = −.11, *t* = −1.28, *p* = .20, *p‐*adj = .27. The same was true for switch RT, which also showed no significant relationship with the rumination questionnaires based on correlation analyses: PTQ: *r* = .06, *t* = 0.71, *p* = .48, *p‐*adj = .55; RRS‐B: *r* = .04, *t* = 0.43, *p* = .67, *p‐*adj = .74; ARS: *r* = .16, *t* = 1.93, *p* = .06, *p‐*adj = .09. Thus, the findings fail to support H5.

However, the correlation analyses did yield significant relationships between DFlex score and all three forms of rumination (supporting H6) and this was confirmed with regression analyses (PTQ: *β* = .39, *t* = 6.04, *p* < .001, *p*‐adj < .001; and RRS‐B: *β* = .35, *t* = 4.72, *p* < .001, *p*‐adj < .001, and ARS: *β* = .42, *t* = 5.57, *p* < .001, *p*‐adj < .001), controlling for gender, hyperacusis, depression and anxiety. The observed relationship between cognitive flexibility and rumination, despite the MAFT indices showing no significant association with rumination, again points to the notion that cognitive and affective flexibility, while interconnected, may represent distinct constructs.

**FIGURE 9:**
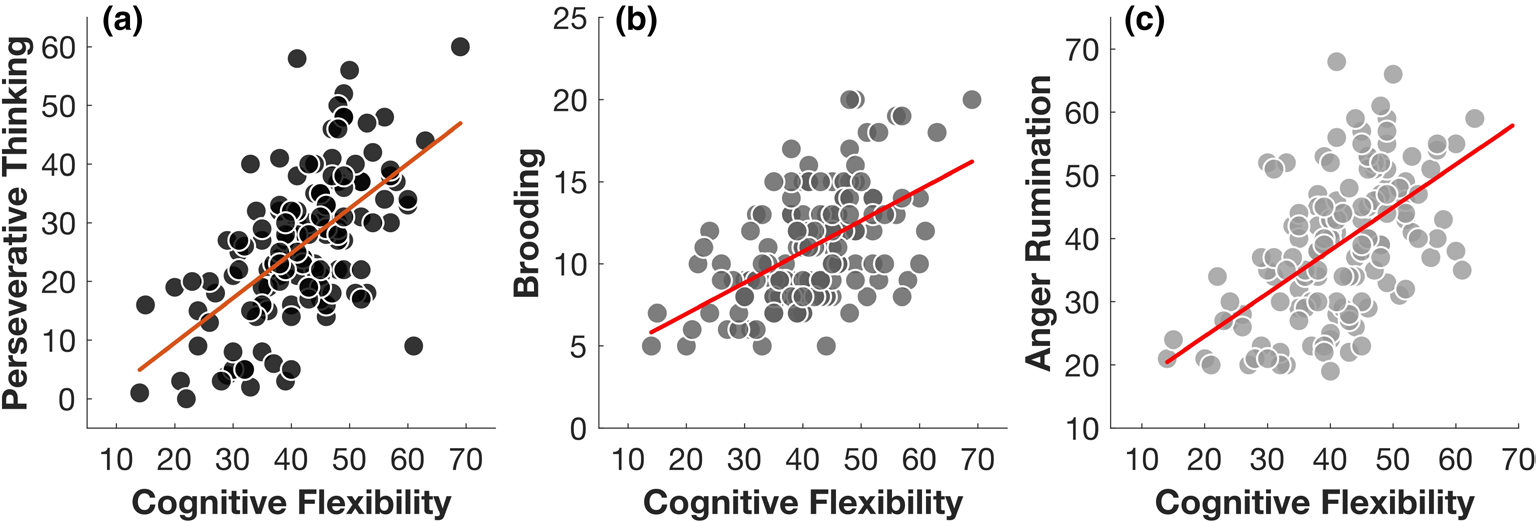
The scatterplots show the bivariate relationships between DFlex, representative of cognitive flexibility and (a) perseverative thinking, (b) brooding and (c) anger rumination. The circles represent individual data points, while the diagonal red lines indicate the line of best fit.

Alternatively, it is also possible that the association between rumination and self‐reported cognitive inflexibility, but not with task‐based affective flexibility, reflects shared method variance among self‐report measures rather than a substantive dissociation. Future research should use multi‐method approaches to clarify whether these constructs are truly separable (Figure 9).

Since significant associations were observed between the S‐Five total score and all three types of rumination (perseverative thinking, brooding and anger rumination) alongside cognitive flexibility, it is reasonable to argue that the interplay between rumination and cognitive flexibility may better explain the variations in S‐Five total. To explore this interaction, three separate regression models were applied with PTQ, RRS‐B, ARS and DFlex as independent (e.g. S‐Five total ~ PTQ + Dflex), and S‐Five total as the dependent variables, while controlling for gender, hyperacusis, depression and anxiety.

Mediation analyses revealed that the relationship between cognitive inflexibility and misophonia severity was significantly mediated by perseverative thinking, brooding and anger rumination, even after controlling for all covariates. Specifically, indirect effects via PTQ (ACME = 0.54, 95% CI [0.13, 1.02], *p* = .008, *p*‐adj = .008), RRS‐B (ACME = 0.57, 95% CI [0.23, 0.99], *p* < .001, *p*‐adj < .001), and ARS (ACME = 0.59, 95% CI [0.22, 1.02], *p* = .002, *p*‐adj = .003) were all statistically significant. It is important to note that due to the exploratory nature of these mediation analyses, we treated them as a distinct family of statistical tests. Thus, we performed FDR correction on generated *p*‐values separately from the main FDR correction for the original set of hypotheses (H1–H6). Across models, approximately 39%–43% of the total effect of cognitive inflexibility on misophonia was accounted for by these ruminative tendencies, highlighting their potential role as cognitive mechanisms linking rigidity to misophonic distress.

## DISCUSSION

In the current work, we extend previous research on cognitive inflexibility in misophonia in several ways. Consistent with hypotheses, we demonstrated that affective flexibility, limited to switch accuracy, was negatively associated with overall misophonia severity, alongside the internalization, impact and outburst factors. We also observed that cognitive inflexibility was significantly associated with overall misophonia severity, and these findings generalized across externalization and outburst, with marginal associations observed for impact and threat but not for internalization factors. Consistent with previous work, we found that cognitive flexibility and affective flexibility were distinct, with no significant correlation between these indices. Furthermore, our findings revealed that various types of rumination, including perseverative thinking, brooding and anger‐related rumination, were associated with misophonia severity and cognitive inflexibility but not affective inflexibility.

Taken together, these findings imply that the mechanisms of misophonia may not be specific to sound triggers. Instead, misophonia appears to involve a complex interplay of cognitive and emotional processes, which may contribute to the heightened sensitivity and exaggerated responsivity to specific auditory or other multisensory stimuli. That is, we observed reduced task‐switching accuracy when faced with emotion‐evoking stimuli that were unrelated to auditory triggers, which suggests broader cognitive‐affective contributors to this disorder, beyond reactions specifically linked to misophonic triggers. Findings also highlight the potential role of maladaptive emotional regulation strategies, such as rumination, in the pathology of misophonia. Overall, our findings emphasize the need to consider cognitive and affective processes in understanding this condition.

### Switching to affective stimuli: greater challenges linked to increased misophonia severity

To provide evidence of affective flexibility deficits in individuals with misophonia, we employed a newly developed task designed to capture affective switching. This task allowed us to explore the relationship between two performance indices, switch accuracy and switch RT, and misophonia severity. As expected, overall misophonia severity and affective flexibility, albeit limited to switch accuracy, were inversely associated and the results held even after controlling for cognitive flexibility to make sure the observed effect was not simply due to general task‐switching (see the Results section for a discussion of the limitations in comparing DFlex and MAFT). However, RT was not related to misophonia, and this is consistent with early evidence that RTs on this task are not specific to affective flexibility but tend to reflect a more general motor response rate across conditions (Allen et al., 2025). The observed diminished affective flexibility associated with misophonia may hamper the ability to effectively switch responding to demands in the face of emotional stimuli. This finding is in line with previous studies which highlight the role of emotion regulation in misophonia (Bagrowska et al., 2022; Barahmand et al., 2023; Cassiello‐Robbins et al., 2020; Dixon et al., 2024; Guetta et al., 2022).

Similar patterns have been revealed in individuals with high autistic traits, overlapping with misophonia symptoms (Ertuerk et al., 2023; Rinaldi et al., 2023), who show greater switch costs (worse accuracy), suggesting potential links between cognitive inflexibility and the expression of autistic traits (Zhou et al., 2024). Two broad, mutually inclusive classes of explanation can be distinguished to account for the increased switch costs observed in individuals with misophonia. First, the Task‐Set Reconfiguration (TSR) Theory (Rogers & Monsell, 1995) posits that switch costs arise from the cognitive effort required to reconfigure a new task set during switch trials, a process reliant on endogenous, top‐down control mechanisms (Zhou et al., 2024). Evidence suggests that cognitive control, a mechanism that facilitates task reconfiguration, is impaired in individuals with misophonia when confronted with trigger sounds (Daniels et al., 2020). In addition, effective cognitive control is crucial for deploying response inhibition when needed, such as restraining automatic but contextually inappropriate behaviours, like anger outbursts in response to trigger sounds. Altered response inhibition related to misophonia has also been observed using the stop‐signal task, revealing a bias towards longer stop‐signal delays (Eijsker et al., 2019). According to this framework, the larger emotion switch costs observed in individuals with misophonia may reflect difficulties in reconfiguring the emotional task set. Second, the Task‐Set Inertia (TSI) Theory (Allport & Wylie, 1999; Meiran, 2008; Waszak et al., 2003; Wylie & Allport, 2000) asserts that switch costs arise from interference caused by residual activation of previous task sets. From this perspective, the larger emotion switch costs in misophonic individuals may indicate greater susceptibility to interference from prior task sets when transitioning to an emotional task. Both task‐set inertia and task‐set reconfiguration are thought to jointly contribute to the increased emotion switch costs (Vandierendonck et al., 2010).

The reduced affective flexibility in misophonia can also be better understood by the neural correlates of misophonia. Inhibition is essential for set‐switching within affective flexibility, facilitating the shift between behaviour, emotion, or cognitive process or task sets by suppressing competing or irrelevant ones and minimizing interference during the transition (Arbuthnott & Frank, 2000). Elevated orbitofrontal cortex (OFC) activity has been documented during successful, as opposed to failed, behavioural inhibition, highlighting its role in regulatory control processes (Deng et al., 2017). In individuals with misophonia, the OFC exhibits hyperactivation specifically in response to trigger stimuli. This heightened activity suggests the critical role of the OFC in facilitating functional synchronization among brain regions, potentially influencing the misophonic ‘behavioural response’ as reflected in behavioural task performance (Cerliani & Rouw, 2020).

### Heightened misophonia severity links to reduced cognitive flexibility

Confirming our behavioural findings and replicating the results of (Simner et al., 2021), individuals with more severe misophonia showed reduced cognitive flexibility. These findings imply a broader contextual rigidity in misophonia, extending beyond specific behavioural responses. Such rigidity has been observed in other overlapping psychiatric and neurodevelopmental disorders (Geurts et al., 2009; Gruner & Pittenger, 2017; Miles et al., 2020; Murphy et al., 2012) with high comorbidity with misophonia (Erfanian et al., 2019; Erfanian & Rouw, 2018; Jager, de Koning, et al., 2020; Rouw & Erfanian, 2018).

Cognitive inflexibility in misophonia may account for heightened sensitivity to norm violations in uncontrollable conditions (Banker et al., 2022), reflecting a diminished ability to adapt to environmental deviations or unpredictability (Miyake et al., 2000) and to flexibly adjust cognitive strategies to manage change (Han et al., 2012). Similar to individuals with autism, those with cognitive inflexibility often resist change and struggle to shift their mental frameworks or expectations, leading to a rigid adherence to established norms (Lacroix et al., 2024). Consequently, when faced with uncontrollable situations that deviate from these norms, their difficulty in adapting results in heightened sensitivity and an amplified reaction to perceived violations (Norena, 2024). This rigidity intensifies discomfort and limits their ability to contextualize or tolerate variability.

Although mental rigidity, attributed to misophonia, is likely a key explanation for the tendency to over‐attend or hyperfocus in response to misophonic triggers (Edelstein et al., 2013; Jager, de Koning, et al., 2020; Simner et al., 2021), it may also represent an underlying pathological mechanism that contributes to broader perceptual and behavioural deficits. To advance the field and explore whether the behavioural symptoms are underpinned by the rigidity or rigidity should be considered per se as a symptom, experimental measures must evolve to reflect mechanistic models of flexibility deficits.

### The severity of misophonia is linked to elevated rumination tendencies

Building on the evidence linking cognitive flexibility and rumination across various psychopathological conditions, we delved into the potential association of misophonia with three forms of rumination (Altan‐Atalay et al., 2022; Davis & Nolen‐Hoeksema, 2000; Genet et al., 2013; Lei et al., 2022; Miles et al., 2023; Owens & Derakshan, 2013). All three forms of rumination were strongly associated with misophonia severity, and these relationships remained significant even after controlling for depression and anxiety. This finding implies that the relationship between rumination and misophonia severity cannot be explained by the presence of these comorbid symptoms. Although rumination is a hallmark feature of depressive and anxiety disorders (Ruscio et al., 2015), both of which frequently co‐occur with misophonia (Erfanian et al., 2018, 2019; Erfanian & Rouw, 2018; Jager, de Koning, et al., 2020; Rosenthal et al., 2022; Rouw & Erfanian, 2018), our findings emphasize the unique connection between rumination and misophonia severity.

Anecdotal reports, consistent with our findings, indicate that individuals with misophonia employ internal coping strategies. These strategies involve cognitive processing of trigger characteristics, anger rumination, and contemplation of emotional responses associated with misophonia, aimed at avoiding or escaping triggers (Dozier & Mitchell, 2023).

### Rumination is linked to cognitive inflexibility yet unrelated to affective inflexibility

Furthermore, findings supporting the link between misophonia and rumination should be interpreted in the context of cognitive flexibility, particularly given its association with all forms of rumination in this study. Of particular relevance is the view that both inflexibility and rumination represent outcomes of emotion dysregulation (Lask et al., 2021; Wang et al., 2025).

First, the inability to mentally switch between cognitive sets may lead to perseveration (Altan‐Atalay et al., 2022; Beckwé et al., 2014; Nolen‐Hoeksema, 2000; Watkins & Brown, 2002). Second, similar to mood disorders, the ruminative tendencies observed in misophonia could reflect disturbances in cognitive switching processes (Piguet et al., 2010), leading to maladaptive emotion regulation strategies that are less effective in the moment (Barahmand et al., 2023; Cassiello‐Robbins et al., 2020; Dixon et al., 2024; Erfanian et al., 2018; Guetta et al., 2022; Rinaldi et al., 2023; Spencer et al., 2023).

It is important to note that we were not able to disentangle the conjoint effects of cognitive flexibility vs. rumination in relation to misophonia. All forms of rumination and cognitive inflexibility overlap in their relationship with misophonia. Previous research supports the bidirectional effects of cognitive flexibility and rumination (De Lissnyder et al., 2012). For instance, in the context of AN, which shows high comorbidity with misophonia, evidence suggests that eating disorder‐specific rumination acts as a mediator between subjective cognitive flexibility and eating disorder symptoms (Miles et al., 2023).

All in all, as we measured multiple forms of rumination and focused on general tendencies of responding to distress rather than misophonia‐specific experiences, our findings demonstrate that a tendency towards perseverative thought in misophonia extends beyond trigger‐related situations. Fortunately, perseverative thought such as rumination can be targeted in treatment through therapy modalities such as CBT (Querstret & Cropley, 2013), which has shown promise in managing misophonia symptoms (Jager, Vulink, et al., 2020).

### Limitations and future directions

While this study advances our understanding of the cognitive‐affective profile of misophonia, certain limitations warrant consideration. The absence of a non‐affective cognitive flexibility task restricts definitive conclusions about whether the observed deficits are specific to affective contexts or reflect broader task‐switching difficulties. However, the inclusion of cognitive flexibility as a covariate in our models provides some reassurance that the associations observed with affective flexibility are not solely attributable to general cognitive flexibility. Nonetheless, future research would benefit from including a non‐affective switching task matched for cognitive demands to more precisely delineate the specificity of these effects. Second, exclusion criteria relied on self‐report and general eligibility screening (e.g. no current psychiatric diagnosis), which may have failed to detect comorbid psychiatric or audiological conditions. Although we controlled for anxiety, depression, and hyperacusis, undetected comorbidities could have influenced results, limiting attribution of effects only to misophonia. Third, our sample combined Prolific participants with individuals recruited through misophonia‐specific newsletters, the latter of whom were likely more symptomatic. This may have inflated severity estimates and introduced sampling bias in correlational findings. Future work could stratify analyses by recruitment source or use matched sampling designs. Fourth, self‐report measures, while validated, remain vulnerable to introspective and social desirability biases. Some analyses were exploratory and should be interpreted with caution pending replication. Fifth, the combination of participants from Prolific and misophonia‐specific newsletters may have biased severity estimates, as the latter group is likely more symptomatic. Future studies should recruit from more uniform or diagnostically verified samples to address this. Finally, while causal relationships cannot be established from the current data, it still remains an open question whether inflexibility and rumination contribute to misophonia or arise from repeated emotional reactivity. These processes may be bidirectional. Longitudinal or experimental studies are needed to clarify their role.

## CONCLUSION

This study provides novel evidence that the severity of misophonia is related to affective inflexibility, cognitive inflexibility and rumination. Affective inflexibility was distinct from cognitive inflexibility and rumination, as no significant association was observed with measures of these constructs. Although causal relationships cannot be inferred from these findings, misophonia may involve not only heightened sensory sensitivity but also disruptions in self‐regulation, such as cognitive inflexibility and rumination, which may either contribute to or arise from misophonic reactivity. Investigating these processes may help clarify whether misophonia constitutes a stand‐alone clinical entity or reflects broader vulnerabilities in regulatory functioning. These findings provide insights into the rich set of affective and cognitive dimensions involved in misophonia. The profile of affective and cognitive difficulties observed here are well‐documented as transdiagnostic risk variables, consistent with the diagnostic overlap of misophonia with conditions such as ASD and OCD. By delineating these characteristics, the study contributes to reducing the risk of misdiagnosis and enhances our understanding of the cognitive and affective processes involved in misophonia. Importantly, this research offers practical implications for clinical practice, encouraging a more targeted approach to treatment protocols that emphasize addressing the cognitive aspects of misophonia.

## AUTHOR CONTRIBUTIONS

**Vivien K. Black:** Conceptualization; investigation; writing – original draft; methodology; validation; writing – review and editing; software; data curation; formal analysis; funding acquisition. **Kenneth J. D. Allen:** Methodology; validation; writing – review and editing; software; data curation; formal analysis. **Hashir Aazh:** Writing – review and editing; supervision. **Sheri L. Johnson:** Funding acquisition; writing – review and editing; project administration; supervision; resources; data curation. **Mercede Erfanian:** Conceptualization; funding acquisition; writing – original draft; methodology; validation; writing – review and editing; visualization; formal analysis; project administration; supervision; resources; data curation.

## Data Availability

All raw and processed data as well as MATLAB and R code for statistical analyses and visualizations are available at https://osf.io/kdrce/.
